# Assessment of Knowledge, Practices, and Attitudes Toward Prostate Cancer and Its Screening Among Men Aged 40 Years and Older in the United Arab Emirates: A Cross-Sectional Study

**DOI:** 10.7759/cureus.75108

**Published:** 2024-12-04

**Authors:** Mahmoud Eladl, Bahaaeldeen Hesham, Saryia Adra, Ahmad Addasi, Mohammad Al Tahawi, Musa'ab Omair, Nafe Alhariri, Mohamed Eladl, Hiba J Barqawi

**Affiliations:** 1 Department of Clinical Sciences, College of Medicine, University of Sharjah, Sharjah, ARE; 2 Department of Clinical Sciences, Mansoura Manchester Program for Medical Education, College of Medicine, Mansoura University, Mansoura, EGY; 3 Sharjah Institute for Medical Research, University of Sharjah, Sharjah, ARE

**Keywords:** male health awareness, prostate cancer, psa, screening, uae

## Abstract

Introduction: Prostate cancer is the second most common cancer in men globally and the fifth greatest cause of cancer-related mortality. In the United Arab Emirates (UAE), prostate cancer has been on the rise due to population aging. However, knowledge deficits and screening barriers do exist because of cultural, social, and psychological factors. The objective of this study is to assess the knowledge, practices, and attitudes of prostate cancer and its screening among men aged 40 years and above in the UAE.

Methodology: A cross-sectional study was conducted between September 5 and October 31, 2024, using a self-administered online questionnaire distributed through social media platforms. Data collected from the questionnaire were analyzed using SPSS version 26 (IBM Corp., Armonk, NY).

Results: A total of 471 responses were analyzed. The median age of participants was 51 ± 12. Most of the respondents were non-healthcare workers with at least a bachelor's degree. Most of the participants were moderately aware of prostate cancer risk factors and symptoms. However, there was a severe lack of awareness of genetic predisposition and racial risk factors. While 80.3% of participants were aware of the role of prostate-specific antigen test in prostate screening, 62.8% recognize the role of a digital rectal examination. The most important barriers to screening were lack of perceived risk, time constraint, and fear of diagnosis.

Conclusion: Significant knowledge gaps and barriers to prostate screening exist. Health education initiatives targeting cultural stigma with the use of culturally appropriate and accurate information through credible online platforms are necessary.

## Introduction

Prostate cancer is a disease that affects middle-aged men. It is the second most common cancer worldwide and the fifth greatest cause of cancer-related mortality [1]. According to the Global Cancer Observatory, prostate cancer posed a significant global health burden in 2022, with more than 1.4 million new cases and over 375,000 deaths reported [1]. While prostate cancer is more prevalent in Western countries, it is on the rise in developing countries, especially in the Middle East [2,3]. Per the United Arab Emirates (UAE) national cancer registry, prostate cancer accounted for 11.9% of cancer cases in 2022 [4].

The prostate-specific antigen (PSA) test is the main method used for the early detection of prostate cancer. However, the use of PSA as a screening tool remains controversial. It often leads to overdiagnosis and overtreatment of otherwise indolent cases, placing patients at risk for treatment-related adverse effects, such as incontinence and erectile dysfunction [5]. These concerns and cultural influences result in diminished participation in screening programs despite efforts to enhance awareness.

Regional prostate cancer awareness campaigns, such as the "Every Voice Matters" campaign, have highlighted the ongoing efforts to address cultural stigmas and improve participation in screening programs [6]. Despite of the UAE health campaigns aimed to encourage prostate cancer screening, especially during November, Men’s Health Awareness Month, participation remains constrained [7]. Men in the UAE, like those in other Middle Eastern nations, frequently forgo screening due to several obstacles, like cultural views on prostate examination as in digital rectal examination (DRE), societal stigmas, and apprehension over diagnosis and treatment [8-13]. Moreover, numerous men in the region possess inadequate awareness regarding the significance of early detection and maintain skepticism concerning the dangers associated with prostate cancer [10]. This fallacy is exacerbated by the cultural hesitance to candidly address male reproductive health concerns, leading to a deficiency in knowledge and a low rate of screening participation.

Considering these issues, the public health implications of prostate cancer in the UAE are anticipated to escalate in the next years, especially due to the nation's aging population. It is essential to implement tailored interventions that tackle the cultural, social, and psychological obstacles to prostate cancer screening. Despite advancements, it is vitally important to implement comprehensive initiatives aimed at teaching men about the advantages and disadvantages of screening while also mitigating the stigma around prostate health. Improving the accessibility of information, increasing awareness, and delivering culturally appropriate health education may result in an increase in participation rates in the screening programs and, consequently, improve the patient's outcomes.

Due to the lack of sufficient studies in the UAE regarding prostate cancer awareness, this cross-sectional study was conducted to evaluate the knowledge, attitudes, and practices regarding prostate cancer and its screening among men aged 40 years and above in the UAE.

## Materials and methods

Study design and target population

An observational, cross-sectional study was conducted to evaluate the knowledge, attitudes, and practices of prostate cancer and its screening among men in the UAE. The inclusion criteria for this study were men aged 40 and above, living in the UAE, who spoke either English and/or Arabic, as those are the only languages spoken by the researchers in the study. Those with a history of prostate cancer, women, and those younger than 40 years of age were excluded. Tourists and visitors to the UAE were excluded from the study. A minimum sample size of 385 was calculated based on 5% marginal error and 50% prevalence, using the following formula: $n=\frac{4p\left(1-p\right)}{SE^{2}}$, where n is the sample size, p is the expected prevalence, and SE is the sampling error. To account for nonresponse, attrition, and incomplete responses, the sample size was increased by 20%, making the minimum sample size required for this study to be 462 participants. The study was approved by the Research Ethics Committee of the Medical Colleges at the University of Sharjah (REC-24-06-11-01-F).

Data collection tool and process

A self-administered questionnaire was developed following a thorough literature review and adapted from multiple validated questionnaires [14-16]. The questionnaire consisted of 43 close-ended and two open-ended questions split into four sections: demographics (13 questions), knowledge and information (20 questions), practices (six questions), and attitudes (six questions), as shown in the Appendix. The questionnaire comprised true or false, multiple choice, and Likert scale questions. Initially, the questionnaire was developed in English and then translated into Arabic, which is the official language of the UAE. Subsequently, a language expert reviewed the translated version, providing feedback to rectify any grammatical errors and ensure linguistic accuracy.

A participant information sheet was presented before starting the study, which specified that filling out the questionnaire indicated consent to participate in the study. The questionnaire was completed online using a link that was shared through various social media methods such as WhatsApp, LinkedIn, and email. The study was conducted between September 5 and October 31, 2024.

Data collection and analysis

Following data collection through Google Forms, the data were imported into Microsoft Excel for initial data cleaning. Subsequently, the data were then analyzed via SPSS Statistics for Windows version 26.0 (IBM Corp., Armonk, NY). The normality of the continuous variables was assessed using both Q-Q plots and the Shapiro-Wilk test. As the data are not normally distributed, the median of continuous variables and interquartile range (IQR) were reported. Valid percentages were reported to account for missing data. Bivariate analyses were performed to identify significant predictors. Statistical tests were performed, including Mann-Whitney U, Kruskal-Wallis, and chi-square tests. A p value of <0.05 was considered to be significant.

## Results

Demographics

A total of 529 participants filled out the form; upon deleting those who did not meet the inclusion criteria, 471 cases were analyzed. The median age of the participants was 51 ± 12. More than half of the participants, 251 (53.3%), had a bachelor's degree, while 88 (18.7%) had a postgraduate degree. The majority of the participants, 407 (86.4%), were married, 353 (74.9%) were other Arabs, and 128 (29.3%) lived in Dubai, followed closely by Sharjah, 133 (28.2%). Most of the participants were in non-healthcare jobs, 380 (80.7%) and had health insurance, 368 (78.1%).

When asked whether the patient or a family member had any type of cancer, only 127 (27.0%) said yes. Of those who said yes, 72/127 (56.7%) were first-degree relatives. The most frequently reported cancer was breast cancer, 27/127 (22.9%), followed by tumors of the digestive system, 23/127 (19.5%). Further demographic details are displayed in Table 1.

**Table 1:** Knowledge of the participants toward prostate cancer and its screening per demographic characteristics (n = 471) The data have been represented as n (%) ^*^p value is calculated using the Kruskal-Wallis test ^**^p value is calculated using the Mann-Whitney test IQR: interquartile range; UAE: United Arab Emirates

Demographic details		n (%)	Knowledge score		p value
			Median	IQR	
Highest degree attained	Middle school or lower	16 (3.4)	19	4	<0.001^*^
	High school	52 (11)	18	2	
	Diploma	64 (13.6)	18	3	
	Bachelor’s degree	251 (53.3)	19	4	
	Postgraduate degree	88 (18.7)	22	4	
Marital status	Single	25 (5.3)	17.5	5	0.18^*^
	Married	407 (86.4)	19	5	
	Divorced	29 (6.2)	19	6	
	Widowed	10 (2.1)	18.5	6	
Nationality	UAE National	59 (12.5)	18.5	3	0.002^*^
	Other Arab	353 (74.9)	20	5	
	Non-Arab	59 (12.5)	18.5	7	
Place of residency	Abu Dhabi	105 (22.5)	21	5	0.004^*^
	Dubai	138 (29.6)	20	5	
	Sharjah	133 (28.5)	20	4	
	Northern Emirates	91 (19.5)	19	4	
Field of work	Healthcare professional	42 (8.9)	23	6	<0.001^*^
	Non-healthcare professional	380 (80.7)	19	4	
	Unemployed (healthcare)	6 (1.3)	22	4	
	Unemployed (non-healthcare)	43 (9.1)	18.5	4	
Health insurance status	Insured	368 (78.1)	20	5	0.96^**^
	Not insured	103 (21.9)	19	3	
Presence of comorbidities	Yes	185 (39.3)	19	4	0.028^**^
	No	286 (60.7)	20	5	
Family history of cancer	Yes	127 (27)	19	5	0.33^**^
	No	344 (73)	20	4.5	
Relationship with the family member with a history of cancer	First degree	72 (56.7)	19	5	0.82^*^
	Second degree	45 (35.4)	19	5	
	Third degree	3 (2.4)	17	-	
	Me/spouse	7 (5.5)	21	2	

Knowledge

The knowledge scores ranged from 9 to 29, with a possible score between 0 and 31. The overall median score was 20 ± 5. Participants with comorbidities had a median score of 19 ± 5, while those without comorbidities had a median of 20 ± 5, a statistically significant difference (p = 0.028) with an effect size of 1.03%. There were also significant differences in the knowledge score of the participants based on their level of education (p < 0.001), nationality (p = 0.002), place of residence (p = 0.004), and field of work (p < 0.001) (Table 1).

The majority of respondents correctly identified that the prostate gland is located below the bladder, 422 (89.6%), produces fluid that is part of semen, 404 (85.8%), and that older men are more likely to develop prostate cancer, 401 (85.1%). However, awareness of genetic and racial risk factors varied. Although 327 (69.4%) were aware that African and Arab men with a family history of prostate cancer are at a higher risk, only 232 (49.3%) correctly recognized that African and Arab men are more commonly diagnosed with prostate cancer than others. Additionally, 360 (76.4%) did not consider race or ethnicity to be a risk factor for prostate cancer, and 117 (24.8%) did not realize that having a father with prostate cancer increases their own risk.

Awareness of prostate cancer screening tools was somewhat limited. While 378 (80.3%) knew that a PSA blood test could be used for screening, only 296 (62.8%) identified that a DRE is also in the assessment of prostate health. Furthermore, 343 (72.8%) correctly acknowledged that neither PSA nor DRE is 100% accurate in detecting prostate cancer. Importantly, 254 (53.9%) thought that prostate biopsy was a blood test rather than a tissue sampling procedure.

**Figure 1:**
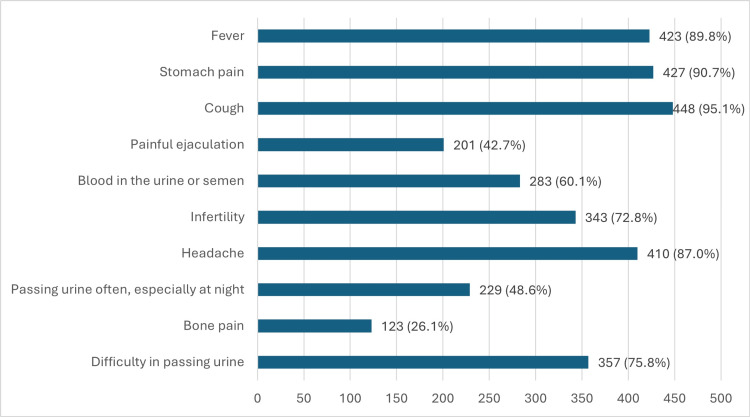
Participants' knowledge of prostate cancer symptoms The data has been represented as n (%)

When asked about signs of prostate cancer, 210 (44.6%) believed that warning signs are always present. Other warning symptoms correctly recognized by participants included difficulty passing urine, 357 (75.8%), and blood in urine or semen, 283 (60.1%). However, only 250 (53.1%) identified lower back pain as a possible sign of prostate cancer, 229 (48.6%) recognized frequent urination, especially at night, and 201 (42.7%) associated painful ejaculation with the disease. On the other hand, some symptoms were incorrectly identified as warning signs: cough, 448 (95.1%); pain in the stomach, 427 (90.7%); fever, 423 (89.8%); headache, 410 (87.0%); and infertility, 343 (72.8%), as shown in Figure 1.

Practices, attitudes, and sources of knowledge

Only 86 (18.3%) and 54 (11.5%) underwent PSA testing and/or DRE, respectively. The majority, 316 (67.1%), reported that they had not been screened. Among those who had been screened, 40/155 (25.8%) had a positive result. When asked about their willingness to get screened, 240 (51.0%) responded negatively. Of those who were willing to undergo prostate screening, the most commonly reported reason was the desire to detect prostate cancer before symptoms arise, 116/231 (50.2%), followed by physician recommendation, 110/231 (47.6%). Interestingly, most of the participants who expressed willingness to be screened did not perceive themselves to be at risk, 26/231 (11.3%). Importantly, those with a family history of cancer were 1.66 (95% CI = 1.102-2.510) times more likely to undergo prostate screening than others (p = 0.017).

Among those who reported barriers to getting screened, the most commonly cited reason was a lack of perceived risk, 90/220 (40.7%), followed by lack of time, 87/220 (39.5%). Fear of a prostate cancer diagnosis was also a factor, 41/220 (18.6%), while cultural beliefs or social stigma were reported by only 29/220 (13.2%).

**Figure 2:**
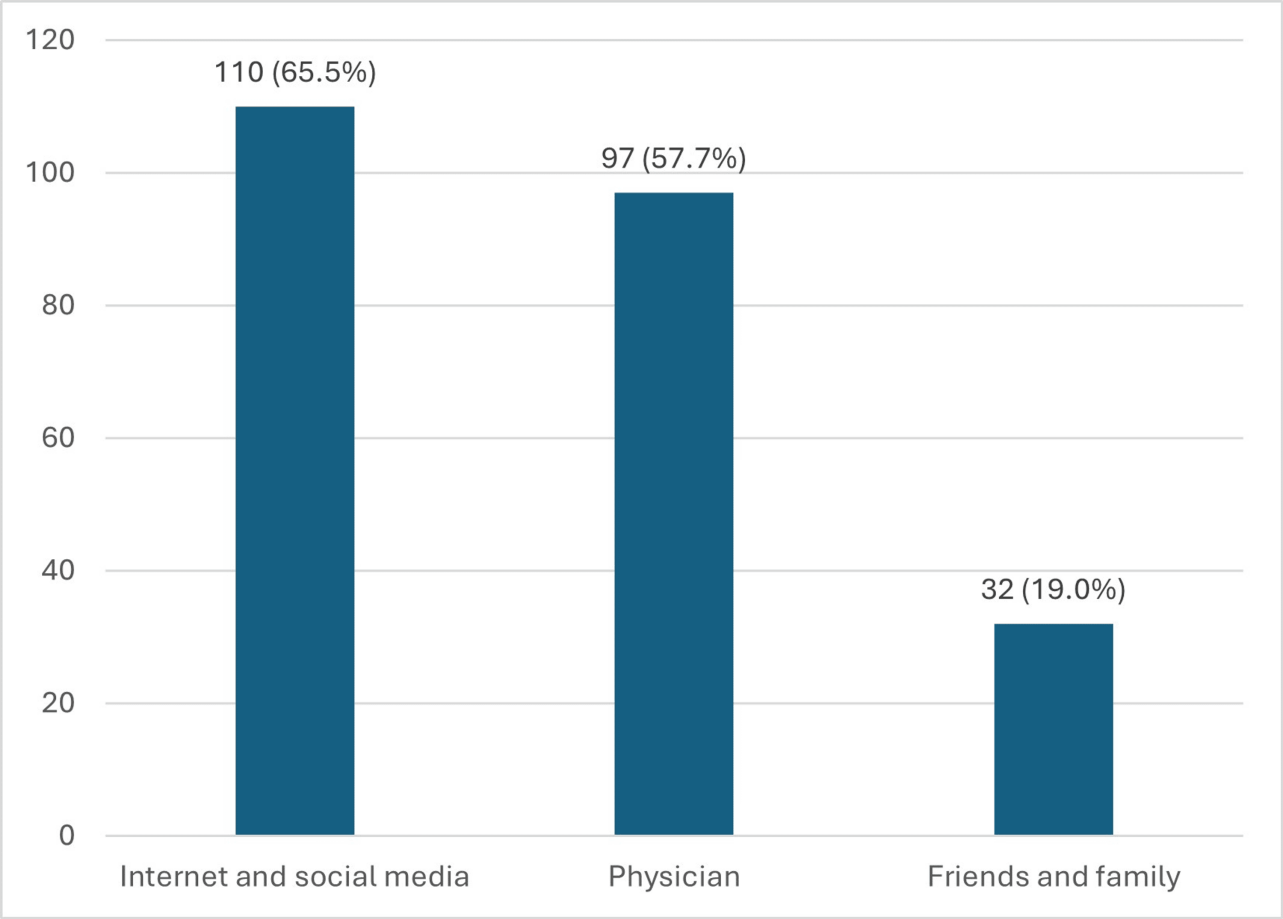
Sources of information on PSA The data has been represented as n (%) PSA: prostate-specific antigen

The internet and social media were the most commonly reported sources of information about PSA testing, 110/168 (65.5%), followed by physicians, 97/168 (57.7%), as displayed in Figure 2. However, most participants, 303 (64.3%), had not received any information about PSA testing. Among those who did receive information, 156/168 (92.9%) found it useful.

Only 35.7% had previously received information about the PSA test, with the remainder indicating a lack of awareness. Among those who had received information about prostate cancer, 42.5% rated the information as "Useful," and 27.2% rated it as "Very Useful."

## Discussion

Although most participants had at least a bachelor’s degree, the overall knowledge was moderate, with significant gaps in knowledge, screening practices, and perception. Additionally, the participants generally demonstrated cultural barriers to screening. The majority of respondents demonstrated good knowledge of the basic anatomy and physiology of the prostate gland. However, knowledge of genetic and racial risk factors is limited, as shown by the fact that less than 50% of respondents correctly identified the increased risk among men of African and Arab descent. This is in concordance with studies conducted in Saudi Arabia and Jordan, where similar knowledge gaps regarding genetic predispositions and their effects on health-seeking behaviors were also emphasized [12,17]. Additionally, a Tanzanian and a Nigerian study showed similar results; importantly, lower levels of knowledge were associated with decreased screening participation [18,19]. These studies suggest the need for culturally appropriate public health initiatives that emphasize the at-risk population and highlight the importance of screening.

This study also highlights the public's misconceptions about prostate cancer screening tools. While most participants were familiar with the use of the PSA test as a screening method for prostate cancer, fewer understood the role of DRE in the assessment of prostate health. Moreover, more than half of the participants incorrectly believed that a prostate biopsy is a blood test. Lack of knowledge of these tools may be influenced by cultural stigmas and discomfort around discussing male reproductive health. These results are in concurrence with other literature reporting that men avoid DRE because of societal stigma and perceived invasiveness [20,21]. As such, targeted public health campaigns are needed to highlight the common misconceptions and to provide clear and scientifically correct information pertaining to prostate screening. Such campaigns would improve the populations' knowledge levels as well as make them more comfortable considering the options of screening for prostate.

The low recognition of prostate cancer symptoms was another major concern. The majority of the participants associated irrelevant symptoms to prostate cancer, such as cough and fever, whereas fewer participants were able to identify lower back pain, frequent urination, and painful ejaculation as possible warning signs of the disease. Other studies in Saudi Arabia similarly reported findings indicating that men tend to confuse symptoms of other common diseases with those of prostate cancer, resulting in a delay in seeking care [12,22]. Therefore, public education about the signs and symptoms of prostate cancer may encourage men to seek medical advice sooner if they are able to correctly recognize the warning signs of the disease, promoting earlier diagnosis and reducing late-stage disease presentation.

Interestingly, only 27% of the participants said they had a family history of cancer, with the majority of those affected being first-degree relatives. Studies have shown that family history influences the perception of risk; still, only 24.8% of the respondents acknowledged their increased risk of prostate cancer if their father was diagnosed with the disease. This finding is similar to other studies, where men with a family history of prostate cancer showed poor awareness of their increased risk, emphasizing the importance of targeted communication with men at familial risk to encourage early screening activities [12,14,23].

Less than 20% and around 10% of participants underwent PSA testing and/or DRE, respectively, highlighting the low engagement rate in prostate cancer screening. When asked about the reasons they did not undergo screening, over half of the participants cited a lack of perceived risk, lack of time, and fear of diagnosis as the main reasons. While those findings are similar to studies conducted in the region, in our study, less than 15% of participants stated cultural beliefs or social stigma as barriers to screening [8-13].

Regarding sources of information for the PSA tests, the internet and social media were the most chosen, followed by physicians. The results highlight the reliance on informal sources of information rather than seeking information from healthcare professionals, which could have contributed to their misconceptions regarding prostate cancer. The above implies a strong need for better communication between healthcare providers and patients on prostate health by organizing awareness campaigns that provide clear information on screening, especially through credible online platforms and social media to bridge this gap and enable the dissemination of information to a broader audience and facilitating better public understanding [12,24,25].

Furthermore, given the UAE's diverse demographic groups, we suggest the need for further studies with higher sample size to validate the findings across a broader population, especially for non-Arabic and non-English speakers as well as for individuals living in the rural and underserved areas, as well as correlating the population's knowledge and awareness of prostate cancer to their socioeconomic status. These studies will help strengthen the interventional efforts and ensure the inclusiveness of various population groups.

Study limitations

As data collection was done via an online questionnaire, two possible limitations arose. First, responder bias is implicated simply because participants are self-reporting their own data without any intervention from the researchers' side. Hence, they might overestimate or underestimate the background knowledge that is being assessed. Second, the lack of accessibility for certain populations with lower levels of computer literacy is an implicated limitation that can significantly impact the generalizability of the results. Additionally, although the questionnaire was available in English and Arabic, a considerable percentage of the population in the UAE are not fluent in either Arabic or English and would rather fill the questionnaire in their native languages. As a solution for the limitations mentioned above, we suggest that further studies should employ a mixed approach by collecting the data both by online survey and in-person to reach the underrepresented groups, including those who are computer illiterates and those who are living in underserved rural areas. Translating survey materials into other languages spoken by expatriates in the UAE, like Urdu, could also enhance inclusivity. Addressing these limitations can improve the generalizability of future findings and inform more targeted interventions.

## Conclusions

There are significant gaps in the knowledge, attitudes, and practices regarding prostate cancer among the studied population in the UAE. The participants' understanding when it comes to screening methods, as well as signs and symptoms of prostate cancer, is severely limited. It was also evident that cultural and practical barriers, including fear of being diagnosed, a lack of perceived susceptibility, and constraints on time, significantly hinder involvement in screening programs. Culturally appropriate educational intervention through the development of accessible online educational content in multiple languages shared on credible online platforms, community outreach programs, as well as training healthcare providers on initiating proactive discussions regarding prostate health through professional development workshops might help in the dissemination of accurate information and reduction of stigma associated with prostate health, hence improving early detection rates and improving the chances of better outcomes for prostate cancer patients in the UAE. Furthermore, we suggest that integrating prostate cancer screening into routine health checks for men over the age of 40, particularly in settings like occupational health clinics and wellness programs, can enhance access and reduce logistical barriers.

## Appendices

**Table 2:** Self-administered questionnaire to assess the knowledge, practices, and attitudes toward prostate cancer and its screening among men aged 40 years and older in the United Arab Emirates UAE: United Arab Emirates; DRE: digital rectal examination; PSA: prostate-specific antigen

No.	Question	Answers
Demographics		
D1	Do you live in the UAE?	Yes
		No
D2	Sex	Male
		Female
D3	Age (please provide age in numbers)	……………………………………….
D4	Highest degree obtained	Middle school or lower
		High school
		Diploma
		Bachelor’s degree
		Postgraduate Degree (MSc, PhD, etc.) or higher
D5	Marital status	Single
		Married
		Divorced
		Widowed
D6	Nationality	UAE National
		Other Arab
		Non-Arab
D7	Place of residency	Abu Dhabi
		Dubai
		Sharjah
		Ajman
		Umm Al Quwain
		Ras Al Khaimah
		Fujairah
D8	Field of work	Healthcare professionals (nurses, doctors, dentists, pharmacists, healthcare administration, etc.)
		Non-healthcare professional
		Unemployed (healthcare)
		Unemployed (non-healthcare)
D9	Do you have health insurance?	Yes
		No
D10	Do you have any long-term medical conditions (such as high blood sugar, high blood pressure, liver or kidney diseases, thyroid abnormalities, etc.)?	Yes
		No
D11	Have you or anyone in your family suffered from cancer? (if no, skip to question K1)	Yes
		No
D12	What is your relationship with that person?	…………………………………………
D13	What type of cancer?	…………………………………………
Knowledge and information		
K1	The prostate gland is a reproductive organ located below the bladder	True
		False
K2	The prostate gland makes some fluid that is part of the semen	True
		False
K3	Older men are more likely to get prostate cancer	True
		False
K4	More African and Arab men are diagnosed with prostate cancer than others	True
		False
K5	African and Arab men who have a family history of prostate cancer are more likely to get it	True
		False
K6	Who do you think is more likely to get prostate cancer (please select all that applies)	Arab men
		African men
		Caucasian men
		Asian men
		Race is not a factor
		I don’t know
K7	Who do you think is more likely to get prostate cancer	Men whose fathers have had prostate cancer
		Men whose fathers have not had prostate cancer
		It doesn’t make any difference
		I don’t know
K8	A PSA blood test can be done to check for prostate cancer.	True
		False
K9	A DRE can be done to check for prostate cancer	True
		False
K10	The only way a man can know for sure if he has prostate cancer is to have a prostate biopsy	True
		False
K11	A prostate biopsy is when a blood test is used to check for protein in the blood	True
		False
K12	Neither the PSA nor DRE are 100% accurate	True
		False
K13	A man can have prostate cancer and have no symptoms	True
		False
K14	The warning signs of prostate cancer are always present	True
		False
K15	Pain often in your lower back could be a sign of prostate cancer	True
		False
K16	Which of the following are warning signs of prostate cancer? (please select all that apply)	Having a hard time passing urine
		Bone pain
		Passing urine often, especially at night
		Headache
		Infertility
		Blood in the urine or semen
		Painful ejaculation
		Cough
		Pain in the stomach
		High temperature
K17	How useful do you think the information that you received on prostate cancer was?	Very useful
		Useful
		Not useful
		Not useful at all
		I haven’t received any information about prostate cancer
K18	Have you ever received information about the PSA test?	Yes
		No
K19	From whom did you receive information about PSA? (please select all that apply)	Mass media
		Physician
		Internet
		Friends/family
		I haven’t received any information about PSA
K20	How useful do you think the information you received on the PSA test was?	Very useful
		Useful
		Not useful
		Not useful at all
		I haven’t received any information about PSA
Practices		
P1	Have you ever gone to the doctor/urologist for prostate problems?	Yes
		No
P2	Which method of screening was used? (please select all that apply)	PSA
		DRE
		I do not know
		I haven’t been screened before
P3	What was the outcome of the screening?	Positive
		Negative
		I haven’t been screened before
P4	Do you have any intention of getting prostate screening soon?	Yes
		No
P5	Why are you planning to get screened? (please select all that apply)	I was advised to do it
		I feel at risk
		I have participated in prevention programs
		To detect prostate cancer before symptoms occur
		I’m not planning to get screened
P6	What are potential barriers that could prevent you from getting screened for prostate cancer? (please select all that apply)	There are no barriers preventing me from getting screened
		I have no information about it
		I was advised against it
		I do not feel at risk
		Lack of time
		Afraid of discovering prostate cancer
		It’s not useful
		Cultural beliefs
		Social stigma
Attitude		
A1	The possibility of developing prostate cancer increases with age	Strongly agree
		Agree
		I don’t know
		Disagree
		Strongly disagree
A2	The PSA test is an invasive laboratory test	Strongly agree
		Agree
		I don’t know
		Disagree
		Strongly disagree
A3	Men without symptoms over the age of 50 must undergo the PSA test	Strongly agree
		Agree
		I don’t know
		Disagree
		Strongly disagree
A4	Prostate cancer screening has side effects that can cause harmful effects on the body	Strongly agree
		Agree
		I don’t know
		Disagree
		Strongly disagree
A5	How worried are you about developing prostate cancer?	Very worried
		Worried
		Not worried
		Not worried at all
A6	How useful is the PSA test in detecting cancer before the symptoms occur?	Very useful
		Useful
		Not useful
		Not useful at all
		I don’t know

## References

[1] Sung H, Ferlay J, Siegel RL, Laversanne M, Soerjomataram I, Jemal A, Bray F (2021). Global cancer statistics 2020: GLOBOCAN estimates of incidence and mortality worldwide for 36 cancers in 185 countries. CA Cancer J Clin.

[2] Sekhoacha M, Riet K, Motloung P, Gumenku L, Adegoke A, Mashele S (2022). Prostate cancer review: genetics, diagnosis, treatment options, and alternative approaches. Molecules.

[3] Kearney G, Chen MH, Mula-Hussain L (2023). Burden of prostate cancer in the Middle East: a comparative analysis based on global cancer observatory data. Cancer Med.

[4] Harbi AZ, Belaila BAB, Shelpai W, Razzak HA (2024). UAE national cancer registry. Cancer Care in the United Arab Emirates.

[5] Tidd-Johnson A, Sebastian SA, Co EL (2022). Prostate cancer screening: continued controversies and novel biomarker advancements. Curr Urol.

[6] (2024). Health forum launches ‘Every Voice Matters’ cancer initiative. Saudigazette.

[7] (2024). Majority of UAE men aren’t checking for cancer symptoms. https://www.clevelandclinicabudhabi.ae/en/media-center/news/majority-of-uae-men-arent-checking-for-cancer-symptoms.

[8] Basamih KA, Alsaedi HM, Alotaibi WK (2024). Exploring the willingness and understanding of digital rectal examinations in assessing anorectal conditions among Saudi patients in the Western region of Saudi Arabia. Cureus.

[9] Humaid Al-Shamsi S, Humaid Al-Shamsi A, Humaid Al-Shamsi M (2023). The perception and awareness of the public about cancer and cancer screening in the United Arab Emirates, a population-based survey. Clin Pract.

[10] Sayan M, Eren AA, Tuac Y (2024). Prostate cancer awareness in the Middle East: a cross-sectional international study. JCO Glob Oncol.

[11] Arafa MA, Farhat KH, Rabah DM (2015). Knowledge and attitude of the population toward cancer prostate Riyadh, Saudi Arabia. Urol Ann.

[12] Jarb AF, Aljuaid AK, Alghamdi SM, Almathami AA, Altawili AA, Alesawi A (2022). Awareness about prostate cancer and its screening in Medina, Jeddah, and Makkah, Saudi Arabia population. Urol Ann.

[13] Boustany J, Abdessater M, Akl H (2021). Prostate cancer awareness in the Lebanese population: a cross sectional national survey. BMC Public Health.

[14] Morlando M, Pelullo CP, Di Giuseppe G (2017). Prostate cancer screening: knowledge, attitudes and practices in a sample of men in Italy. A survey. PLoS One.

[15] Gift S, Nancy K, Victor M (2020). Assessment of knowledge, practice and attitude towards prostate cancer screening among male patients aged 40 years and above at Kitwe Teaching Hospital, Zambia. Afr J Urol.

[16] Owens OL, Tavakoli AS, Rose T, Wooten NR (2019). Development and psychometric properties of a Prostate Cancer Knowledge Scale for African American men. Am J Mens Health.

[17] Arafa MA, Rabah DM, Wahdan IH (2012). Awareness of general public towards cancer prostate and screening practice in Arabic communities: a comparative multi-center study. Asian Pac J Cancer Prev.

[18] Awosan KJ, Yunusa EU, Agwu NP, Taofiq S (2018). Knowledge of prostate cancer and screening practices among men in Sokoto, Nigeria. Asian J Med Sci.

[19] Bugoye FC, Leyna GH, Moen K, Mmbaga EJ (2019). Knowledge, perceived risk and utilization of prostate cancer screening services among men in Dar Es Salaam, Tanzania. Prostate Cancer.

[20] Kirby M, Merriel SW, Olajide O (2024). Is the digital rectal exam any good as a prostate cancer screening test?. Br J Gen Pract.

[21] Teoh M, Lee D, Cooke D, Nyandoro MG (2023). Digital rectal examination: perspectives on current attitudes, enablers, and barriers to its performance by doctors-in-training. Cureus.

[22] Almuhanna AM, Alshammari S, Alsalman HK (2018). Awareness of prostate cancer, screening and methods of managements in a hospital in Riyadh, Saudi Arabia. Egypt J Hosp Med.

[23] Mofolo N, Betshu O, Kenna O, Koroma S, Lebeko T, Claassen FM, Joubert G (2015). Knowledge of prostate cancer among males attending a urology clinic, a South African study. Springerplus.

[24] Welch V, Petkovic J, Pardo Pardo J, Rader T, Tugwell P (2016). Interactive social media interventions to promote health equity: an overview of reviews. Health Promot Chronic Dis Prev Can.

[25] Neiger BL, Thackeray R, Van Wagenen SA, Hanson CL, West JH, Barnes MD, Fagen MC (2012). Use of social media in health promotion: purposes, key performance indicators, and evaluation metrics. Health Promot Pract.

